# A Novel Method for Accurate Quantification of Split Glomerular Filtration Rate Using Combination of Tc-99m-DTPA Renal Dynamic Imaging and Its Plasma Clearance

**DOI:** 10.1155/2021/6643586

**Published:** 2021-03-13

**Authors:** Xiaoxi Pang, Fei Li, Shan Huang, Cheng'en Wang, Tao Zhang, Zihao Hu, Hao Cheng, Xinchen Tao, Wenrui Chang

**Affiliations:** ^1^The Second Hospital of Anhui Medical University, Hefei, China; ^2^Beijing Hospital, Beijing, China

## Abstract

**Purpose:**

To precisely quantify split glomerular filtration rate by Tc-99m-DTPA renal dynamic imaging and plasma clearance in order to increase its consistency among doctors.

**Methods:**

Tc-99m-DTPA renal dynamic imaging was performed according to the conventional radionuclide renal dynamic imaging by five double-blinded doctors independently and automatically calculated split GFR, namely, gGFR. Moreover, the conventional radionuclide renal dynamic imaging was assessed to only outline the kidney, blank background, and automatically calculated split GFR, gGFR′. The total GFR value of patients, tGFR, was obtained by the double-plasma method. According to the formula, Precise GFR (pGFR) = gGFR′/(gGFR′ + gGFR′) × tGFR. The precise GFR value of the divided kidney, pGFR, was calculated. The Kendall's *W* test was used to compare the consistency of gGFR and pGFR drawn by five physicians.

**Results:**

According to Kendall's *W* consistency test, Kendall's coefficient of concordance was 0.834, *p* = 0.0001 using conventional method. The same five doctors used blank background again and the same standard Gates method to draw the kidneys, which automatically calculated gGFR′. Using input formula, the pGFR was calculated and Kendall's *W* consistency test (Kendall′s coefficient of concordance = 0.956, *p* = 0.0001).

**Conclusion:**

The combination of Tc-99m-DTPA renal dynamic imaging combined with the double-plasma method could achieve accurate split GFR, and because of the omission of influence factors, the consistency of pGFR obtained by different doctors using this method was significantly higher than that of conventional Tc-99m-DTPA renal dynamic imaging.

## 1. Introduction

Accurate renal GFR is critical for the clinical decision-making of hydronephrosis or renal cancer [1]. Inulin clearance rate is recognized as the gold standard for GFR measurement. However, this method is time-consuming, laborious, and expensive, which is difficult to be applied in clinical practice as well as in scientific research. At present, biochemical indicators or combined with Tc-99m-DTPA radionuclide renal dynamic imaging are often used to evaluate renal function, but there are obvious deficiencies in both laboratory and imaging examinations. For example, laboratory biochemical indicators have obvious lag and cannot obtain the divided renal function. GFR determination by dual-plasma method was highly consistent with inulin clearance rate, and the former procedure was simpler and more acceptable to patients. Therefore, this method has been recommended as the gold standard for GFR determination by the American Nuclear Medicine Association and named as true GFR (tGFR). tGFR has also been recommended by the International Society of Nephrology as a reference index for clinical and scientific research [2]. Ultrasonic, intravenous pyelography (IVP), and radionuclide renal dynamic imaging have many influencing factors, and their objectivity, repeatability, and accuracy are often questioned.

At present, there is no simple and accurate method to quantify the split glomerular filtration rate. The purpose of this study was to combine and complement radionuclide renal dynamic imaging with double-plasma method to explore a new accurate quantitative method for the split glomerular filtration rate in patients with hydronephrosis. This study was approved by the institutional ethical committee of our hospital (NO. YX2021-006 (F1)).

## 2. Materials and Methods

### 2.1. General Information

66 patients with hydronephrosis who were admitted before operation at the Second Hospital of Anhui Medical University were included in this study. There were no restrictions on age and gender among the subjects. Patients meeting the following conditions were included in this study: ① ultrasonography or CT diagnosis of hydronephrosis. This study retrospectively included 41 males and 25 females. The average age of subjects was 51.69 ± 15.19 years old.

### 2.2. Double-Plasma Sampling Method

The same technician took 5 mL of venous blood from the contralateral elbow of each patient at 2 h and 4 h after injecting Tc-99m-DTPA with renal dynamic imaging, respectively, and administered heparin anticoagulation. The serum was separated by centrifuging at 1500 g for 15 min. One mL of plasma was accurately extracted from each pipette, and the radioactivity counts of P1 and P2 were measured with radioimmunogamma counter for 60 s. The counts of radioactive P1 and P2 were substituted into GFR = [Dln (P1/P2)/(T2 − T1)] exP [(T1lnP2) − (T2lnP1)]/(T2 − T1), and the total GFR value, namely, tGFR, is automatically calculated.

### 2.3. Gates Method for Radionuclide Kidney Dynamic Imaging

The Tc-99m-DTPA kit was purchased from Jiangsu Institute of Atomic Energy Medicine with the approval document H20013118, and radiochemical purity was >95%. GE Infinia Hawkeye 4 single photon emission computed tomography with low energy and high resolution collimator was adopted to select 140 keV energy peak, window width 20%, and matrix 64 × 64. The patient was fully hydrated (300-500 mL of regular drinking water) 20-30 min before the examination, and the height and weight were measured and routinely recorded. The patient was then placed in the supine position with the probe placed in the lower back, and the SPECT field of view included both the kidneys and the bladder. Immediately after intravenous injection of 5 mCi Tc-99m-DTPA, images were acquired in front and rear positions of the dual probe. A total of 30 frames were collected at 1 frame/2 s immediately after injection, and 20 frames were collected at 1 frame/60 s. Afterwards, an empty syringe was collected for 6 s to obtain the residual drug count.

**Figure 1:**
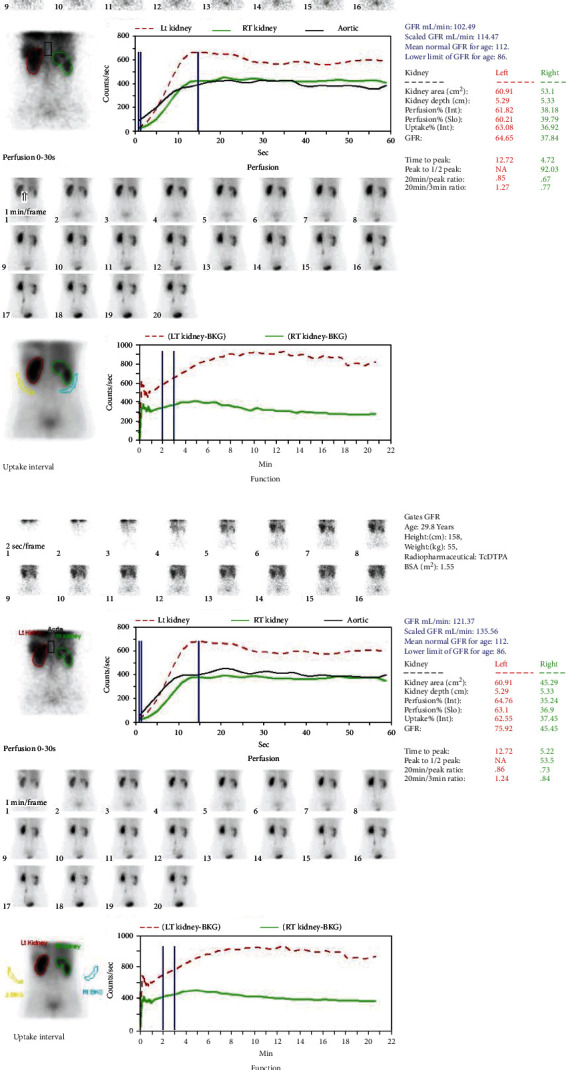
(a) The kidney and background were delineated according to the standard Gates method, and the GFR was automatically calculated. (b) Blank background, residual standard Gates method, delineation of kidney, and automatic calculation of gGFR′.

Five attending physicians who were blinded to the patient details used independent ROI technology with the areas of interest double kidney outline and background (Figure 1), and input intravenous injection of Tc-99m-DTPA radioactive count and the patient's height and weight in the computer to automatically generate double kidney time-radioactive curve, and calculate the double kidney GFR, standardization, and body surface area; the unit is mL·min^−1^·(1.73 m^2^)^−1^.

### 2.4. Accurate Quantification of Renal GFR

The original data of the nuclide kidney dynamic imaging was independently transferred to the postprocessing workstation by each physician who was double-blinded. The professional software was used for processing. After the same conventional operation, points of renal gGFR _left_′ and the split renal gGFR _right_′ were automatically calculated. According to the following formula, accurate GFR of kidney segmentation was automatically calculated (Formula 1): accurate GFR of left kidney: pGFR_left_ = gGFR_left_′/(gGFR_left_′ + gGFR_right_′) × tGFR and accurate GFR of right kidney: pGFR_right_ = gGFR_right_′/(gGFR_left_′ + gGFR_right_′) × tGFR.

### 2.5. Statistical Analysis

The SPSS 19.0 statistical software was used for statistical analysis of the data. The measurement data conforming to the normal distribution was expressed as (*x* ± *s*). The correlation of double-plasma sampling method GFR with uric acid, creatinine, urea nitrogen, and cystatin C was analyzed by Pearson's correlation and linear regression. The Gates method and the consistency of pGFR were applied to Kendall's *W* test by different physicians. A *p* value <0.05 was considered statistically significant.

## 3. Results

### 3.1. Characteristics of the Patients' Information

All patients involved were diagnosed as hydronephrosis by ultrasonography or CT scan. The tGFR in 66 patients was 89.62 ± 46.57 mL/min. The result of gGFR left evaluated by five physicians, respectively (*n* = 66): 41.56 ± 17.04, 43.76 ± 17.74, 45.68 ± 19.41, 40.94 ± 16.45, and 44.84 ± 21.75 mL/min, and the gGFR right: 44.67 ± 17.65, 46.04 ± 18.15, 42.79 ± 16.63, 49.87 ± 20.83, and 44.72 ± 20.97 mL/min. The result of pGFR left evaluated by five physicians, respectively: 46.45 ± 32.04, 44.53 ± 32.44, 45.93 ± 30.63, 46.90 ± 33.46, and 48.39 ± 35.74 mL/min, and the pGFR right: 43.20 ± 31.60, 45.08 ± 31.41, 43.68 ± 30.80, 42.72 ± 32.13, and 41.22 ± 32.15 mL/min.

### 3.2. GFR of Double-Plasma Method and Results of Biochemical Examination

The tGFR in 66 patients was 89.62 ± 46.57 mL/min; the creatinine was 85.59 ± 31.32 *μ*mol/L; the cystatin C was 0.83 ± 0.45 mg/L; the uric acid was 324.95 ± 118.71 *μ*mol/L, and the urea nitrogen was 6.32 ± 2.17 mmol/L.

**Figure 2:**
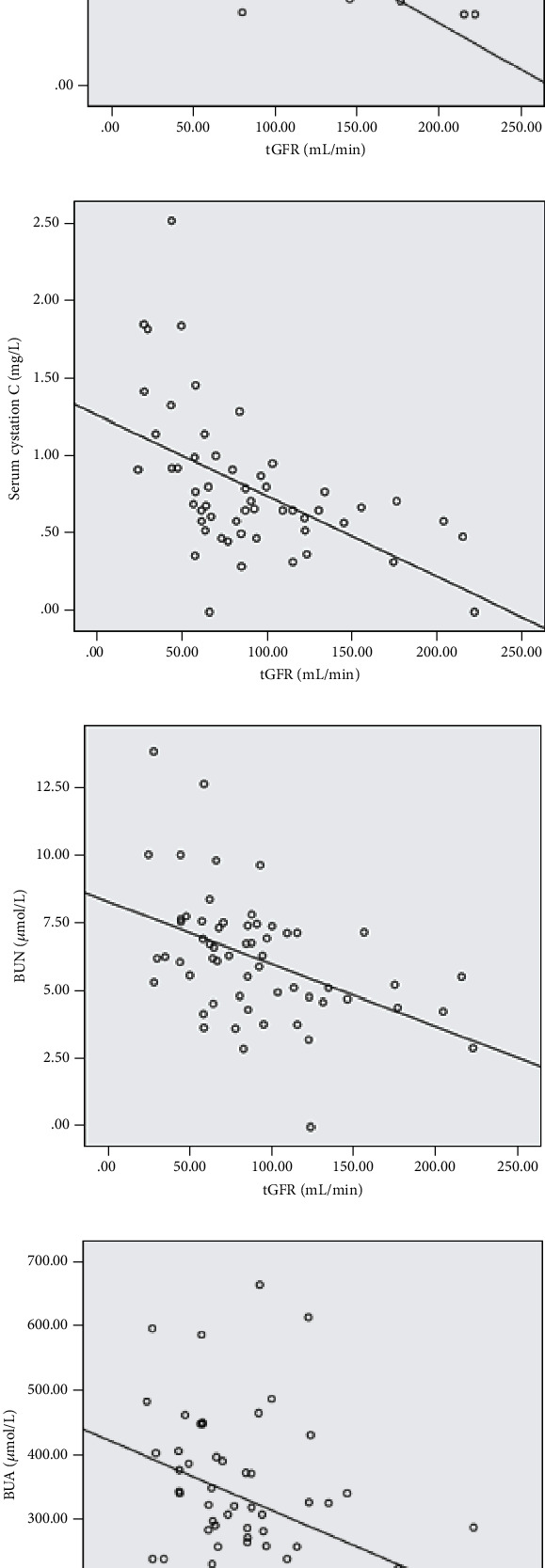
GFR of double-plasma method showed moderate negative correlation with serum creatinine, cystatin C, urea nitrogen, and uric acid.

GFR of double-plasma method showed moderate negative correlation with serum creatinine, cystatin C, urea nitrogen, and uric acid. The correlation coefficients (*r*) were -0.692, -0.527, -0.454, and -0.424, respectively; the coefficient of determination (*R*^2^) were -0.479, 0.278, 0.206, and 0.172, and the *p* values were 0.0001, 0.0001, 0.0001, and 0.001, respectively (Figure 2).

### 3.3. The Consistency of GFR Measured by Conventional Gates Method

Five double-blinded doctors independently used the standard Gates method to outline the background and the kidney and calculated each kidney GFR. The result was 43.46 ± 19.08 mL/min. The results of the five doctors were checked for consistency by Kendall's *W* test. Kendall consistency coefficient of this group, Kendall's *W* = 0.834, *p* = 0.0001, which showed that the background results drawn by the five doctors were consistent.

### 3.4. Precisely Split GFR (pGFR) and Its Consistency

The above five double-blinded doctors independently automatically calculated each kidney gGFR _left_′ and gGFR _right_′, entered formula 1, and automatically calculated pGFR. The result was 53.78 ± 28.16 mL/min, with the exception that the background was not delineated, and other operations were the same as normal operations. The consistency of the pGFR results of the five doctors was tested by Kendall's *W* test. Kendall consistency coefficient of this group, Kendall's *W* = 0.956, *p* = 0.0001, which showed that the background results outlined by the five doctors had a high consistency.

## 4. Discussion

Inulin clearance is recognized as the gold standard for GFR, but this method is complicated and difficult to promote in clinical practice. The Tc-99m-DTPA multiplasma method has a correlation coefficient of 0.99 with inulin clearance, which is often used as a reference standard for determining renal GFR [3], but it has also failed to achieve widespread clinical acceptance due to the long examination time. The Tc-99m-DTPA double-plasma method has extremely high consistency with the multiple-plasma method, the correlation coefficient is 0.97-0.996, the average deviation is 2.8 mL/min [4], and compared with inulin clearance or the multiple-plasma method, it has better clinical acceptance. In clinical practice, biochemical indicators such as serum creatinine, cystatin C, uric acid, and urea nitrogen are more often used to reflect the state of renal function, but these indicators are affected by many factors, and numerous clinical studies have confirmed that these indicators work best when renal function is significantly impaired. In addition, none of the above methods can obtain points of renal GFR. Radionuclide renal dynamic imaging is currently the only clinical method that can reflect points of renal GFR and urinary tract information through multiple parameters [5]. It is more sensitive and reliable than other methods [6–8] and is widely used to evaluate renal function.

The method of quantification of renal glomerular filtration rate (GFR) by combining conventional radionuclide renal dynamic imaging and double-plasma method reported in this article can avoid the multiple influencing factors of renal dynamic imaging, as well as significantly improve the ROI of different doctors. The method shows consistency of the scored renal GFR and more importantly can obtain accurate points of renal GFR. This method is very useful for evaluating residual renal function in patients with hydronephrosis or renal cancer and can provide an objective basis for urologists to perform nephrectomy and the extent of resection.

### 4.1. The Correlation between Double-Plasma Method and Biochemical Indexes

Creatinine clearance is closely correlated with inulin clearance [9] but is not as exact as inulin clearance in estimating GFR, since a small quantity of creatinine is secreted by the renal tubules [10]. The serum creatinine concentration is affected by factors such as age, gender, muscle composition, and diet. Some drugs and endogenous substances can also interfere with the results. The serum creatinine level will increase only when GFR is reduced to 50%.

The results of this study showed that tGFR was moderately negatively correlated with secreted creatinine, cystatin C, urea nitrogen, and uric acid (*r* = −0.692, -0.527, -0.454, -0.424, *p* < 0.01), and the coefficient of determination *R*^2^ was -0.479, 0.278, 0.206, and 0.172, respectively. Hence, although these biochemical indicators can reflect the state of renal function to a certain extent, serum creatinine, cystatin C, urea nitrogen, and uric acid cannot accurately reflect the renal function, especially the points of renal function to estimate GFR. This is also consistent with previous research results.

This study on the correlation between tGFR and the abovementioned biochemical indicators found that the correlation with tGFR from high to low was serum creatinine, cystatin C, urea nitrogen, and uric acid.

### 4.2. The Consistency of Standard Gates Method

Tc-99m-DTPA scintigraphy is the most widely used method for estimation of GFR in clinical work [11]. However, numerous clinical studies have confirmed that radionuclide renal dynamic imaging is affected by many factors, such as the delineation of the kidney and the background area of interest (ROI), the attenuation coefficient of the radiation emitted by the radionuclide in the body, the net injection dose, the quality of the projectile injection, the kidney depth, and SPECT hardware equipment [12–15], resulting in unstable accuracy and repeatability, and in the case of severe hydronephrosis, the Gates method may significantly overestimate renal GFR.

In this study, five double-blinded doctors independently used the standard Gates method to delineate the background and the kidneys and calculate the points of renal GFR. The results of the five doctors were tested for consistency by Kendall's *W* test, and the group's Kendall's coefficient of concordance = 0.834, *p* = 0.0001, which showed high consistency between the five doctors and the automatically calculated results. There remained a 16.4% difference in the results between different doctors, that is, the renal GFR scored by the Gates method could not accurately divide kidney GFR. Numerous studies have tried to optimize the influencing factors of the ROI delineation method required by the Gates method to further improve the consistency of the ROI delineation of different doctors and obtain accurate renal GFR, but these issues have remained methodologically unresolved.

### 4.3. Accurate Quantitative Results and Consistency of Points of Renal Function

The double-plasma method is recognized as the gold standard for GFR and is commonly used in clinical and scientific research, but it cannot obtain the function of each renal GFR. Tc-99m-DTPA renal dynamic imaging is currently the only method to obtain renal GFR, but its results are affected by many factors, as described above. Although numerous optimization studies have been conducted on the influencing factors of renal dynamic imaging, there is no simple and accurate quantitative method for points of renal function.

In this study, the advantages of points of renal function could be obtained with the accurate total GFR quantification of double-plasma method and renal dynamic imaging. By organically combining the two, five double-blinded doctors independently obtained accurate points of renal GFR, and the consistency of their results was tested by Kendall's *W*. The Kendall's coefficient of concordance Kendall's *W* = 0.956, *p* = 0.0001, which indicated extremely high consistency in the background results outlined by the five doctors, and was also significantly higher than the consistency of the results obtained by the conventional standard Gates method.

The results of this study showed that the double-plasma method combined with Gates renal dynamic imaging can obtain accurate points of renal GFR, and this method has extremely high consistency between the results drawn and calculated by different doctors. This method should be validated in further multicenter studies in different units.

## Data Availability

All data included in this study are available upon request by contact with the corresponding author.
